# Insight into the molecular mechanism of the transposon-encoded type I-F CRISPR-Cas system

**DOI:** 10.1186/s43141-023-00507-8

**Published:** 2023-05-16

**Authors:** Amnah Alalmaie, Saousen Diaf, Raed Khashan

**Affiliations:** 1 Department of Pharmaceutical Sciences, Philadelphia College of Pharmacy, Saint Joseph University, Philadelphia, PA 19131 USA; 2 Department of Pharmaceutical Sciences, Division of Pharmaceutical Sciences, Long Island University, Brooklyn, NY 11201 USA

**Keywords:** Transposon-guided CRISPR, Review, CRISPR Cas, Structural and Molecular Mechanism, TniQ Activation

## Abstract

CRISPR-Cas9 is a popular gene-editing tool that allows researchers to introduce double-strand breaks to edit parts of the genome. CRISPR-Cas9 system is used more than other gene-editing tools because it is simple and easy to customize. However, Cas9 may produce unintended double-strand breaks in DNA, leading to off-target effects. There have been many improvements in the CRISPR-Cas system to control the off-target effect and improve the efficiency. The presence of a nuclease-deficient CRISPR-Cas system in several bacterial Tn7-like transposons inspires researchers to repurpose to direct the insertion of Tn7-like transposons instead of cleaving the target DNA, which will eventually limit the risk of off-target effects. Two transposon-encoded CRISPR-Cas systems have been experimentally confirmed. The first system, found in Tn7 like-transposon (Tn6677), is associated with the variant type I-F CRISPR-Cas system. The second one, found in Tn7 like-transposon (Tn5053), is related to the variant type V-K CRISPR-Cas system. This review describes the molecular and structural mechanisms of DNA targeting by the transposon-encoded type I-F CRISPR-Cas system, from assembly around the CRISPR-RNA (crRNA) to the initiation of transposition.

## Background

CRISPR stands for “clustered regularly interspaced short palindromic repeats” [1]. The CRISPR system is a vital part of the bacterial immune system; it protects bacteria by cutting the DNA of invading viruses [1]. CRISPR sequences have two components: the CRISPR array (which includes palindrome-alternating conserved sequences of genetic code 'repeats' separated by 'spacer' sequences) and Cas genes encoding Cas proteins [2]. CRISPR-Cas systems can generally be classified into two distinct classes based on the organization of Cas proteins responsible for processing and adaptation [3, 4]. Class 1 utilizes multi-Cas effector molecules ( Effector is Cas proteins that mediate a specific effect) bound together with mature crRNA, whereas Class 2 CRISPR-Cas system consists of only one [3].

Researchers have discovered that the class 2 CRISPR-Cas systems can be repurposed and utilized as a gene-editing tool to cut any DNA [5, 6]. Type II CRISPR-Cas9, which belongs to class 2 CRISPR-Cas systems, was the first type used to edit the genome [5]. The crRNA guides the Cas9 nuclease to the double-stranded DNA and cleaves both strands. Cas9 then cuts the complementary strand by introducing double-stranded breaks in the target DNA [5]. Although this technique is efficient, it has a significant drawback: the off-target effect, in which crRNA directs Cas9 to DNA strands with similar but not identical sequences. As a result, Cas9 can cleave the wrong target DNA sequence, even if there is a mismatch between the crRNA and its complementary DNA at one or more positions [7, 8]. Target DNA double-strand breaks caused by Cas9 can be repaired by natural mechanisms such as nonhomologous end joining (NHEJ) and homology-directed repair (HDR) [9]. The NHEJ mechanism involves the ligation of the DNA break ends irrespective of the DNA sequence, causing an insertion/deletion ("indel") mutation; in contrast, homology-directed repair requires a DNA homologous template to precisely incorporate a new DNA sequence [10]. Recently, significant progress has been made in the CRISPR-Cas system to control the repair processes triggered by DNA breaks and to improve the accuracy of CRISPR-Cas tools. A recent bioinformatic analysis revealed the presence of a short CRISPR locus containing a Tn7-like transposon gene in place of the Cas endonuclease gene within the bacterial genome [11]. These transposon-encoded CRISPR-Cas systems can potentially bypass double-strand breaks induced by DNA cleavage and instead integrate Tn7 transposons at specific sites within the target DNA [12–14]. Two studies confirmed the existence of a transposon-encoded CRISPR-Cas system [12, 13]. The first study found that the *Vibrio cholera* cascade complex type I-F variant lacks Cas3, a nuclease used for DNA degradation [12, 15]. This cascade complex has been linked to a transposition protein known as TniQ to direct transposition to a specific site in the genome [12, 15]. Another study found that the V-K variant of *Scytonema hofmannii* lacks a residue that allows Cas12 to typically perform DNA cleavage in CRISPR-Cas type V [13].

This review provides an overview of the transposon-encoded CRISPR-Cas system. It highlights the structural and molecular mechanisms of DNA targeting by the transposon-encoded type I-F CRISPR-Cas system. We first briefly describe the classical CRISPR-Cas system, its classification, and its major limitations as a gene-editing tool. This discussion is followed by a brief description of the Tn7 transposon and Tn7-like transposon families. The following section provides an overview of experimentally validated transposon-encoded CRISPR-Cas systems, emphasizing the steps required to integrate the Tn7 transposon into a target DNA using the transposon-encoded type I-F CRISPR-Cas system. The following section covers experimentally confirmed mutations affecting DNA binding, TniQ dimerization, and RNA-guided DNA integration efficiency. We briefly discuss recent improvements in the transposon-encoded CRISPR-Cas system as a gene-editing technology.

### Classical CRISPR-Cas System

The CRISPR-Cas system is a widespread adaptive immune system found in archaea and bacteria [16]. Three stages characterize CRISPR-Cas immunity. The first is spacer acquisition, also known as "adaptation," followed by pre-crRNA processing, and finally, the interference stage [16, 17]. At the spacer acquisition stage, Cas1 and Cas2, and sometimes Cas4, seize a segment of the target DNA "protospacer" and insert it at the 5′ end of a CRISPR array [16, 17]. The CRISPR array is transcribed into a long transcript known as "pre-crRNA" bound by Cas proteins and processed into mature, small crRNAs, each containing a spacer and a segment of the repeat sequence in the pre-crRNA processing stage [16, 17]. The final stage is interference: the mature crRNA bound by Cas proteins scans the DNA for a protospacer adjacent motif (PAM) sequence [16, 17]. Once the PAM sequence is located, base pairing between the crRNA spacer and complementary DNA protospacer occurs, followed by subsequent cleavage by a dedicated nuclease domain [16, 17]. It is worth mentioning that each Cas protein has a different function, as shown in Table 1.

**Table 1:**
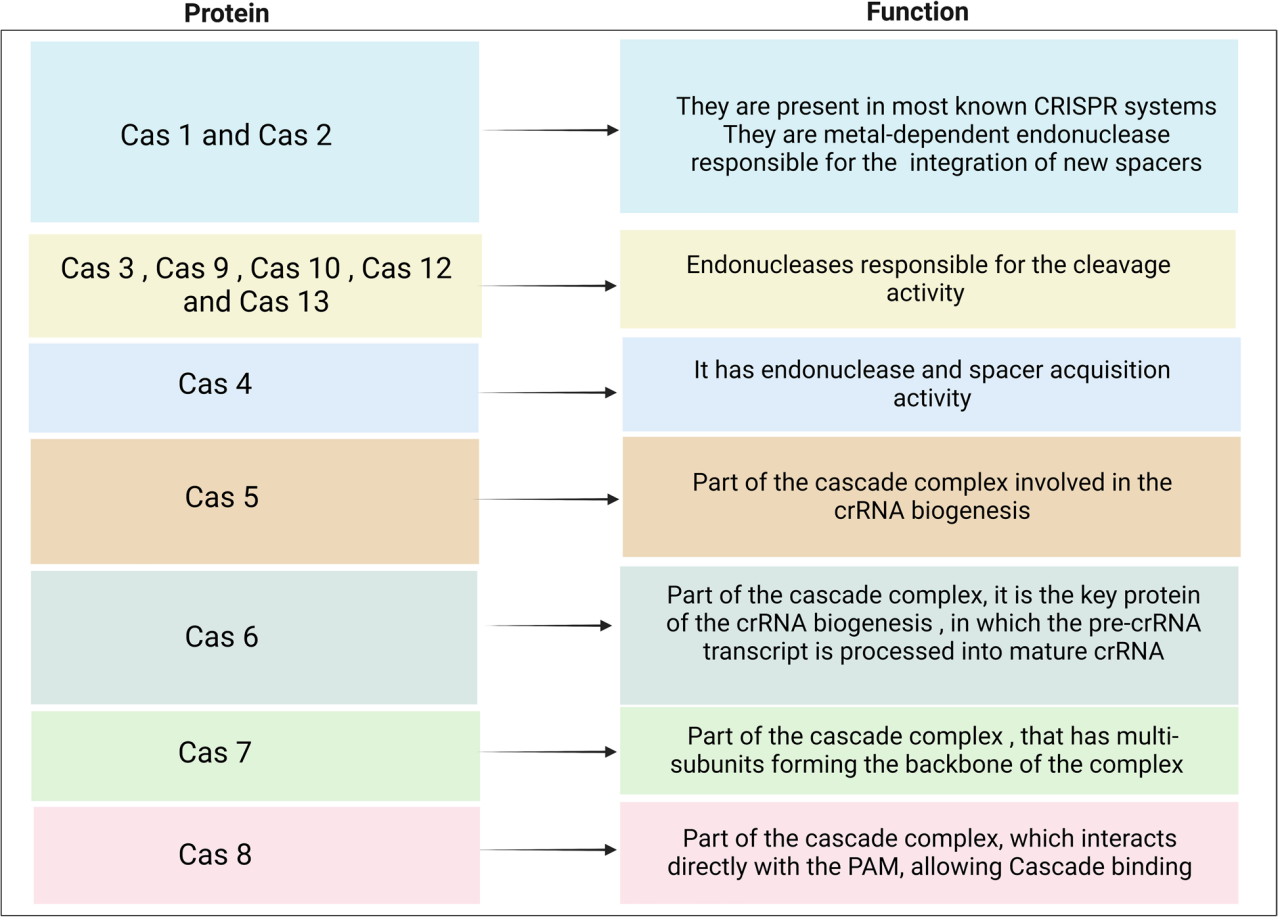
Cas proteins and their function. Created with BioRender.com

The CRISPR-Cas systems can be classified into two main classes: six types and 21 subtypes, as shown in Table 2 [3, 4, 18].

**Table 2:**
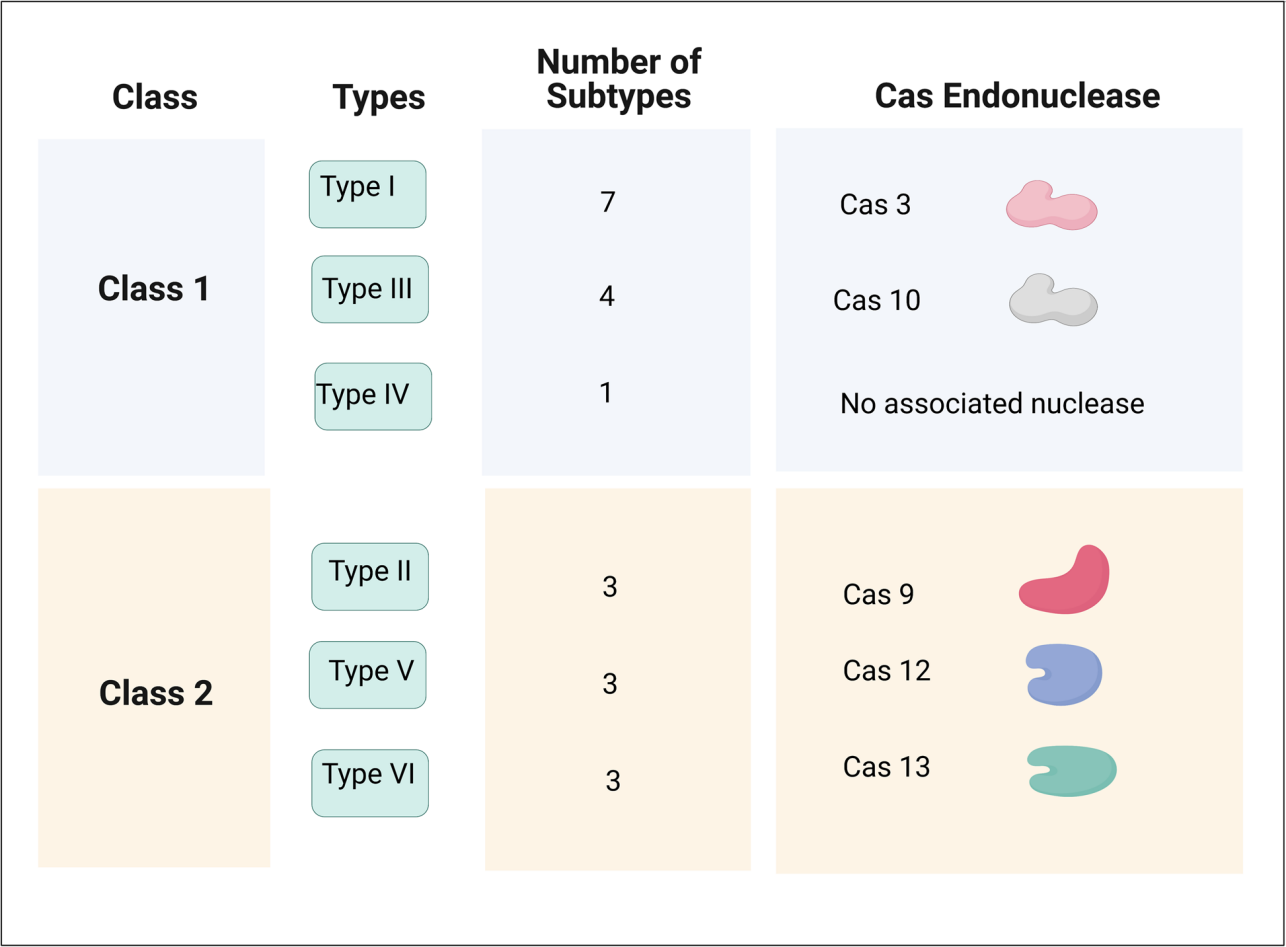
General classification of CRISPR-Cas system. Created with BioRender.com

Class 1 utilizes a multi-protein complex ( known as the cascade complex) and is divided into three types (type I, type III, and type IV) and 12 subtypes [3, 4, 19]. Type I is the most predominant and diverse group of endogenous CRISPR-Cas systems, accounting for 81% of CRISPR-Cas systems [4, 20]. Type I CRISPR-Cas systems are classified into I-A to I-G subtypes, with the most prevalent subtype I-F [4]. Type I systems use a cascade complex containing Cas3, Cas5, Cas6, Cas7, and Cas8 to guide the crRNA to the complementary strand and, subsequently, the target cleavage [21, 22].

Type III CRISPR-Cas systems are classified into four subtypes (III-A through III-D), characterized by the presence of a cascade complex similar to that found in type I; however, Cas 10 is the hallmark gene responsible for target cleavage [21]. The components of the cascade complex vary among subtypes, and several variants of type I systems lack the adaptation genes and genes responsible for DNA cleavage [21]. What is interesting about Type III is its ability to target and cleave both RNA and DNA [21]. No nuclease has been found in type IV, and its function has not been experimentally investigated [4].

Class 2 CRISPR-Cas is characterized by the presence of a single protein that performs all the multi-component tasks in class 1 [19]. The simplicity of the Class 2 system provides a potential for genome-editing applications. There are three types: type II, type V, and type VI, with signature nucleases Cas9, Cas12, and Cas13, respectively [5, 20]. Type II is divided into three subtypes (II-A, II-B, and II-C), all of which share Cas9 as the signature gene responsible for target cleavage [23, 24]. The main difference between subtypes is the size of the Cas9 gene [23, 24]. There are three subtypes under the type V, and VI CRISPR-Cas Class 1 utilizes a multi-protein complex (known as the cascade complex) and is divided into three types (type I, type III, and type IV) and 12 subtypes; class 1 [3, 4, 19]. CRISPR-Cas9 technology has been used successfully for many excellent studies, such as the generation of a CRISPR-based cancer model to understand the molecular details of cancer pathogenesis. Another excellent example is the use of CRISPR-Cas9 to treat sickle cell disease [25, 26]. Systems (V/VI-A, V/VI-B, and V/VI-C), where the hallmark genes are Cas12 and Cas13 [27, 28]. Recently, Cas12 has proven to be an attractive candidate for many applications, such as the use of engineered Cas12 to reproduce the early progression of (human) atherosclerosis in a rat model [29].

As mentioned, Cas nucleases induce double-strand breaks (DSBs) in the target DNA, which are repaired by natural repair mechanisms such as homology-directed repair and nonhomologous end-joining [5, 7, 8]. Transposon-encoded CRISPR-Cas systems bypass the introduction of toxic double-strand breaks in the target DNA, excluding the necessity of the repair mechanisms [12, 15]. Before reviewing the two experimentally confirmed transposon-encoded CRISPR-Cas systems in more detail, the following section briefly describes the Tn7 transposon.

### Tn7-Transposonand Tn7-Like Transposon Family

Transposons are widespread across species and genomes, accounting for nearly half of the human genome. Transposons can move freely within a genome by inserting their own genetic information into host genomes in the presence of transposases [14, 30, 31]. The most crucial feature of transposases is that they catalyze a complete DNA integration reaction without the necessity for either homology-directed repair or nonhomologous end-joining repair mechanisms, which means that the integration process does not introduce broken DNA ends to be repaired [12, 13]. There are two transposition pathways; the first pathway is a replicative transposition, where the transposable segment is copied to the new site leaving the original site intact, known as the copy and paste transposition [32–35]. The second pathway is a conservative transposition, known as cut-and-paste transposition, where the transposable segment is excised from the original location and translocated to a new site via a mobile plasmid [36]. Tn7 is a bacterial transposon that uses the machinery of five transposition proteins, TnsA, TnsB, TnsC, TnsD, and TnsE, as shown in Table 3 [30, 35].

**Table 3:**
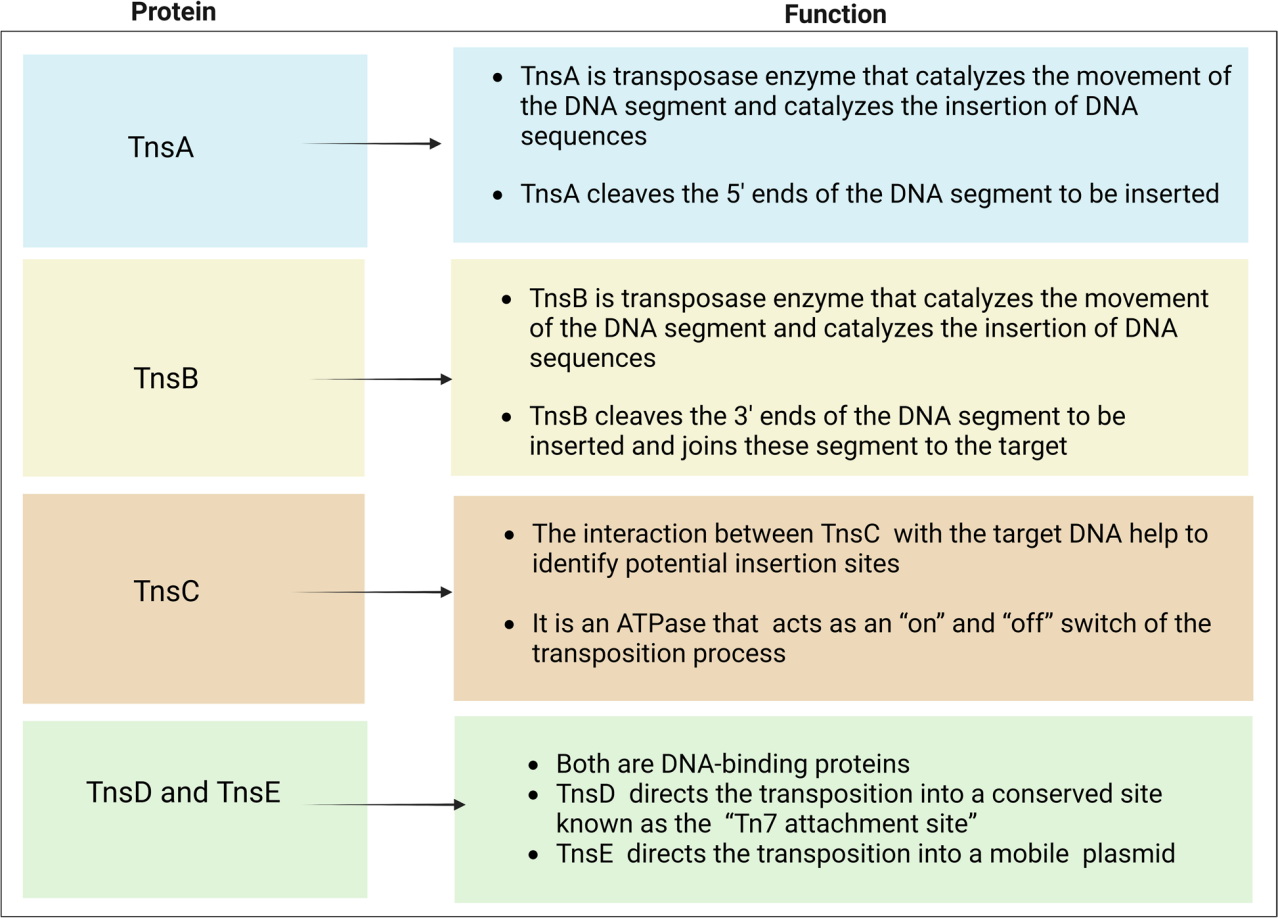
The machinery of the transposition proteins of the Tn7 transposon. Created with BioRender.com

TnsA and TnsB are transposase enzymes that catalyze the movement and integration of DNA sequences [30, 37]. TnsC is an ATPase protein that communicates with TnsA and TnsB to catalyze DNA integration [30, 38]. TnsD and TnsE are target DNA-binding proteins controlling the transposition pathway [30, 35]. The copy-and-paste transposition pathway uses TnsD, which targets the transposition to a specific site in bacteria known as the Tn7 attachment site (attTn7) [32–34]. The cut-and-paste transposition pathway uses TnsE, which directs transposition into a mobile plasmid to facilitate nonspecific horizontal gene transfer [36]. A related transposable element called Tn7-like transposon contains homologues of the Tn7 transposon, in which TnsA, TnsB, TnsC, and TnsD are common between Tn7 and Tn7-like families; however, a recent bacterial genome survey showed that the Tn7-like family could utilize different target site selector proteins instead of TnsE or TnsD [30, 39–41]. Recent studies on Tn7-like transposons have shown that TnsE is replaced with a variant of the type I-F or V-K CRISPR-Cas system [11–13].

### Transposon-Encoded-CRISPR-Cas Systems

The presence of short CRISPR arrays similar to those found in nuclease deficient type I-B, type I-F, and type V-K CRISPR-Cas systems has been reported in several bacterial Tn7-like transposons [11–13]. In these transposon-encoded CRISPR-Cas systems, Cas1 and Cas2 genes, which are responsible for spacer acquisition, are absent, in addition to Cas3 and Cas12 genes, which are responsible for target DNA cleavage [11–13]. The lack of Cas1 and Cas2 limits spacer acquisition, whereas the absence of nucleases renders them inactive in target DNA cleavage [11]. The collaboration between the nuclease-deficient CRISPR-Cas systems and the transposition components of the Tn7 Like family enables these systems to use the guide RNA to direct the Tn7 transposon to be integrated into other sites in the genome [12, 13, 15, 42–45]. RNA-guided DNA integration neither introduces a double-stranded break in the DNA nor depends on the host DNA repair machinery to repair the crack, making the transposon-encoded CRISPR-Cas system a promising candidate for gene editing [12]. Interestingly, the transposon-encoded CRISPR-Cas system allows the insertion of large DNA fragments (up to 10 kb in length) in the genome [46].

#### Type I-F and I-B CRISPR-Cas System Variants Linked to Tn7-Like Family

**Fig. 1:**
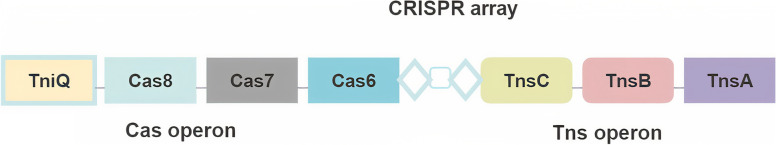
Overall architecture of the *V. cholerae* TniQ–cascade complex [12] Created with BioRender.com

Sternberg’s group showed that the transposition mechanism of the *Vibrio cholera* Tn6677 transposon (VcTn6677) is based on collaboration between the transposition components of the Tn7-like transposon and the nuclease-deficient type I-F CRISPR-Cas system [12, 15]. The *Vibrio cholera* Tn6677 transposon is a complex consisting of two main components: the cascade (CRISPR-associated complex for antiviral defense), which consists of Cas6, six subunits of Cas7, fused Cas8/5 (simply Cas8), and transposition subunits (TnsA, TnsB, TnsC, and TniQ (TnsD-like) [12, 15]. This complex is called INTEGRATE system (INsert transposable elements by guide RNA-assisted targeting) [12, 15]. The Tns terminal operon comprises TnsA, TnsB, and TnsC genes, whereas the TniQ gene (TnsD-like) is present within the Cas operon and not within the Tns operon [12], as shown in Fig. 1

**Fig. 2:**
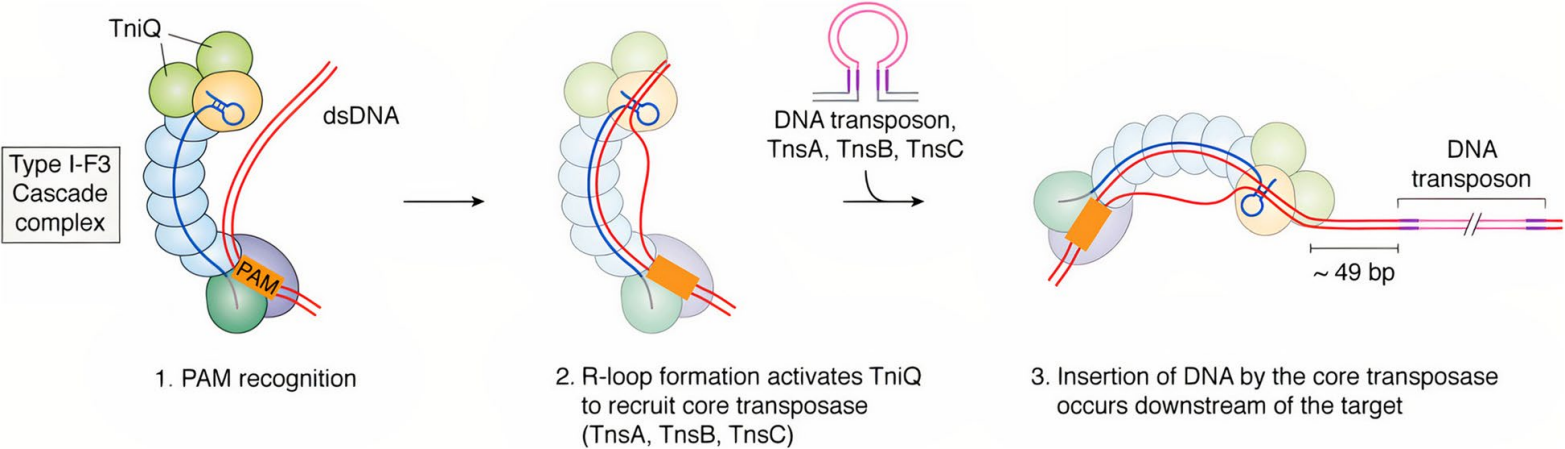
RNA-guided DNA-transposition process [47]. 1-Cas8 of the INTEGRATE system recognizes PAM sequences in double-stranded target DNA. 2- crRNA invades the dsDNA target to form RNA: DNA hybrid with the complementary strand, displacing the non-complementary strand to form the R-loop structure. Subsequently, TniQ, bound to the PAM distal end of the DNA-bound cascade complex, is expected to recruit TnsC, TnsA, and TnsB to activate RNA-guided DNA transposition

DNA binding occurs in three significant steps, as confirmed in four independent studies [15, 42, 44, 45]. First, the cascade components assemble around the crRNA. Then, the cascade/crRNA binds to the TniQ protein [15, 42, 44, 45]. The third step involves binding the cascade/crRNA-TniQ complex to the target DNA to initiate transposition [15, 42, 44, 45]. The following steps can conclude the overall transposition process: the cascade/crRNA-TniQ complex recognizes the target site in DNA, and TniQ recruits TnsC, which eventually recruits TnsA/B-loaded DNA transposons to insert the transposon into the new site [15, 42, 44, 45], as shown in Fig. 2. The transposition occurs 47–51 bp downstream of the cascade target site flanked by a 5-bp target site duplication [12, 15]. The INTEGRATE system has an impressive on-target accuracy, with 99% of the transpositions occurring at precise locations [12].

#### Type V-K CRISPR-Cas System Variant Linked to Tn7-Like Family (ShCast)

Type V-K CRISPR-Cas system variants are closely associated with transposons of the Tn5053 family [13]. Tn5053 is a transposon family that includes TnsB, TnsC, and TniQ but lacks TnsA [13]. This variant CRISPR system contains a single-protein CRISPR-Cas, Cas12, lacking the residues that usually allow target cleavage in type V CRISPR-Cas systems, making it incapable of cleavage [13]. The bacterial *Scytonema hofmannii* Tn7-like transposon is linked naturally to type V-K CRISPR-Cas system variants (known as the ShCast system) [13].

**Fig. 3:**
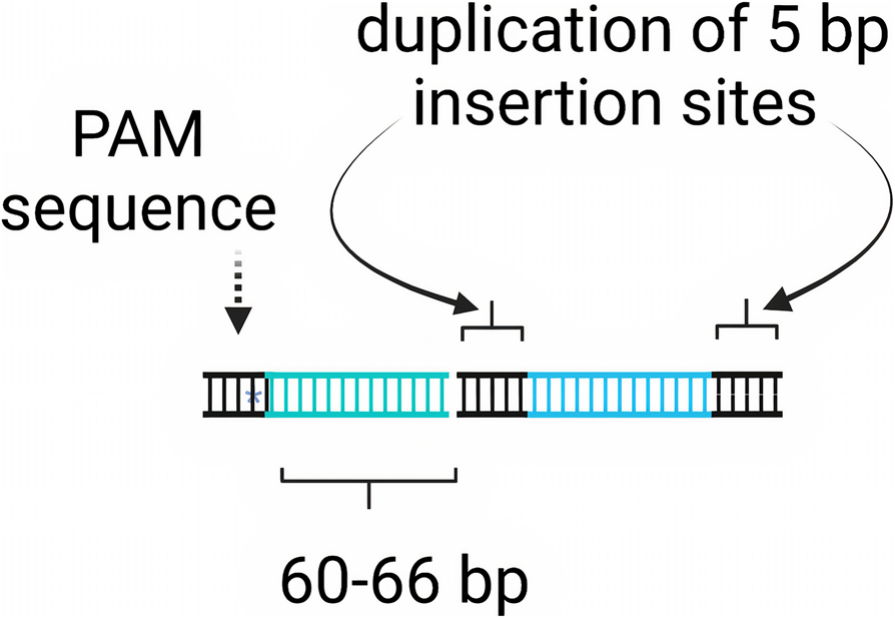
Model for RNA-guided DNA transposition in the ShCAST system [13]. ShCAST mediates the insertion of DNA 60–66 bp downstream of the PAM, resulting in the duplication of 5 bp insertion sites. Created with BioRender.com

As mentioned earlier, the shCAST complex lacks the functional TnsA, making the transposition not only a simple insertion but also a fusion of the donor plasmid [13]. Typically, TnsA and TnsB work together to completely excise the DNA transposon from one site and insert it into the target site, where TnsA cleaves one DNA strand and TnsB cleaves the other DNA strand, leading to the complete excision of the transposon from its original site [13, 37]. The transposition occurs 60–66 bp downstream of the PAM sequences in the target DNA, resulting in the duplication of 5 bp insertion sites [13], as shown in Fig. 3. Interestingly, an engineered variant of Tn5053- like CRISPR systems encoding TnsA/B fusion has been designed [48]. This newly developed system is fully capable of RNA-guided DNA transposition via simple insertion with high target specificity [48]. A recent bioinformatics search reported the presence of new families of CAST-encoded Type I-C and Type IV CRISPR-Cas systems [49].

### Structural and molecular mechanism of the Tn6677 -encoded type I-F CRISPR-Cas System (The INTEGRATE System)

#### Steps of RNA-guided DNA Transposition Process

1. Cas6 of the cascade complex catalyzes pre-crRNA maturation, a critical step in the cascade assembly.

**Fig. 4:**
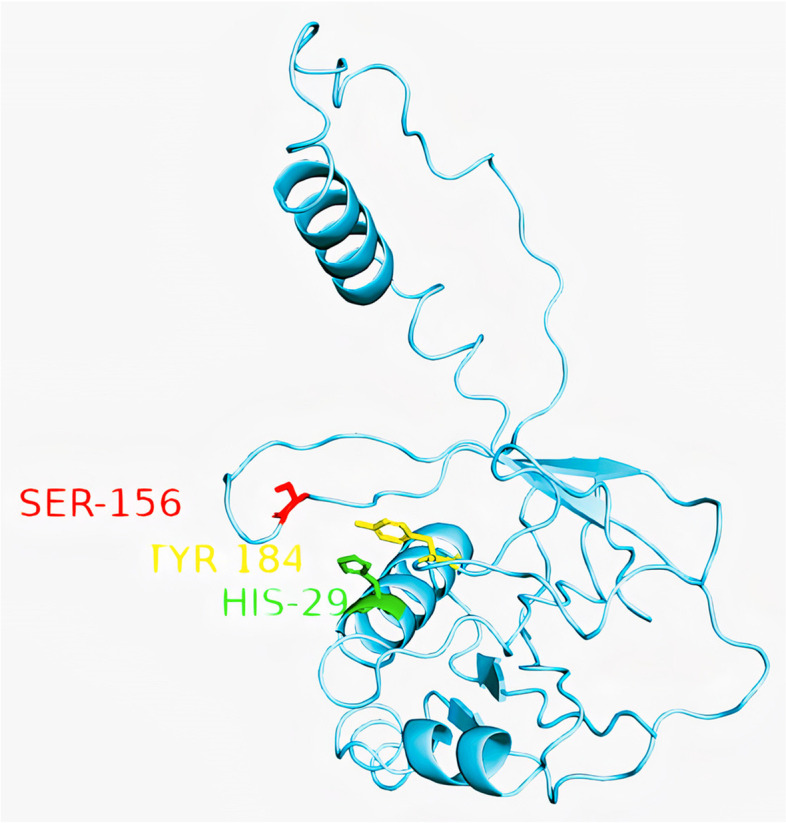
Key residues of Cas6 responsible for the maturation of pre-crRNA into a mature crRNA [45]. Adopted from PDB code: 6LNB https://www.rcsb.org/structure/6LNB. The figure was created with PyMol software [55]

Pre-crRNA maturation is essential in the cascade complex assembly and DNA transposition [45, 50–53]. Cas6 transforms pre-crRNA into mature crRNA by cleaving immature crRNAs to form a stable hairpin structure in each repeat [45, 50–53]. The mature crRNA comprises a full spacer separated by a short repeat-derived 50 handle and a 30-stem loop [54]. After cleavage, Cas6 remains bound to 30 stem-loop structures of crRNA because of its high affinity, acting as a scaffold for the remaining proteins [54]. Mutation of critical residues involved in crRNA cleavage (His29, Ser156, and Tyr184) prevents crRNA maturation and shows no cascade assembly or DNA transposition [12, 45, 50, 52], as shown in Fig. 4.

2. Assembly of the cascade complex around the 60-nucleotides mature crRNA forms the cascade/crRNA complex.

**Fig. 5:**
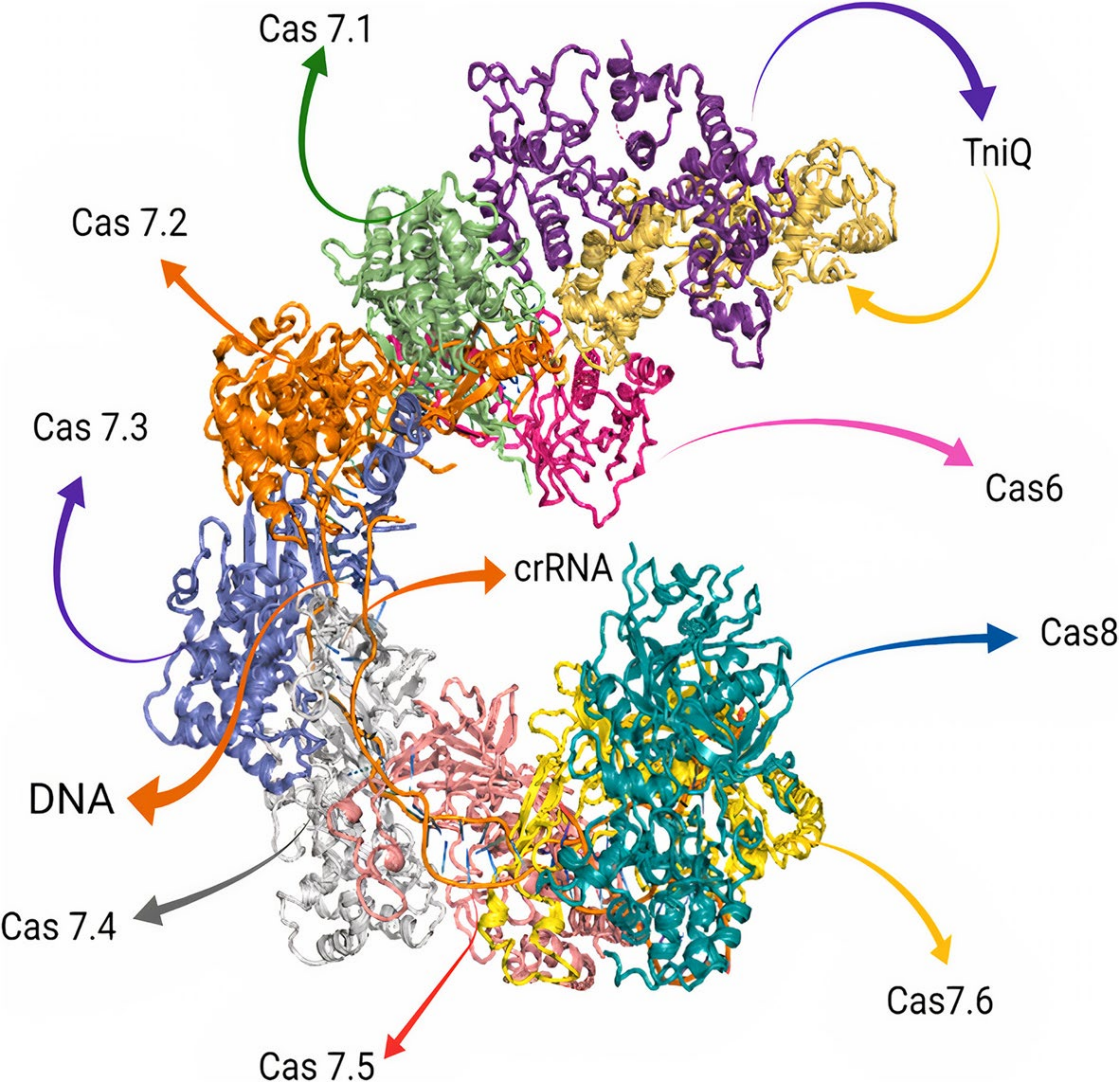
Vc-Cascade-TniQ structure, which adopts a helical (G) architecture where Cas6 forms the head of the complex, six Cas7 subunits form the backbone of the complex, Cas8 forms the tail of the complex in addition to the TniQ dimer. Each monomer of TniQ interacts with the head of the cascade mainly via Cas6 and Cas7.1 to form a head-to-tail TniQ dimer [15]. Adopted from PDB code: 6PIJ https://www.rcsb.org/structure/6PIJ The figure was created with PyMol software [55]

The cascade complex of *Vibrio cholera* consists of (Cas6, six Cas7 subunits, and Cas8) assembled around a 60- nucleotide-crRNA in a helically twisted "G" shape [15, 42, 44, 45]. The arginine-rich α-helix of Cas6 interacts with the negatively charged phosphate groups in the major groove of crRNA at the 3′ stem-loop, forming the head of the cascade complex, whereas Cas8 binds to the 5’ handle of the crRNA, forming the tail of the cascade complex [15, 42, 44, 45]. Cas7 subunits form the backbone of the complex around the crRNA, in which the backbone is capped by the binding of Cas6 and terminated by the binding of Cas8 [15, 42, 44, 45]. The crRNA is oriented in such a way as to place the "backbone" region in the middle and to connect the head to the tail via the interaction between Cas8 and Cas6 [15, 42, 44, 45], as shown in Fig. 5. One of the conserved features in CRISPR-Cas type I is the unique architecture of Cas7 backbone where the ‘thumb’ of each Cas7 subunits folds over the top of the crRNA to create a kink in the crRNA; after five nucleotides, the sixth nucleotide will be flipped out to be on the side (a periodic "5 + 1" pattern) [15, 42, 44, 45, 55].

3. Binding of Cascade/crRNA Complex to TniQ.

#### TniQ Structure

**Fig. 6:**
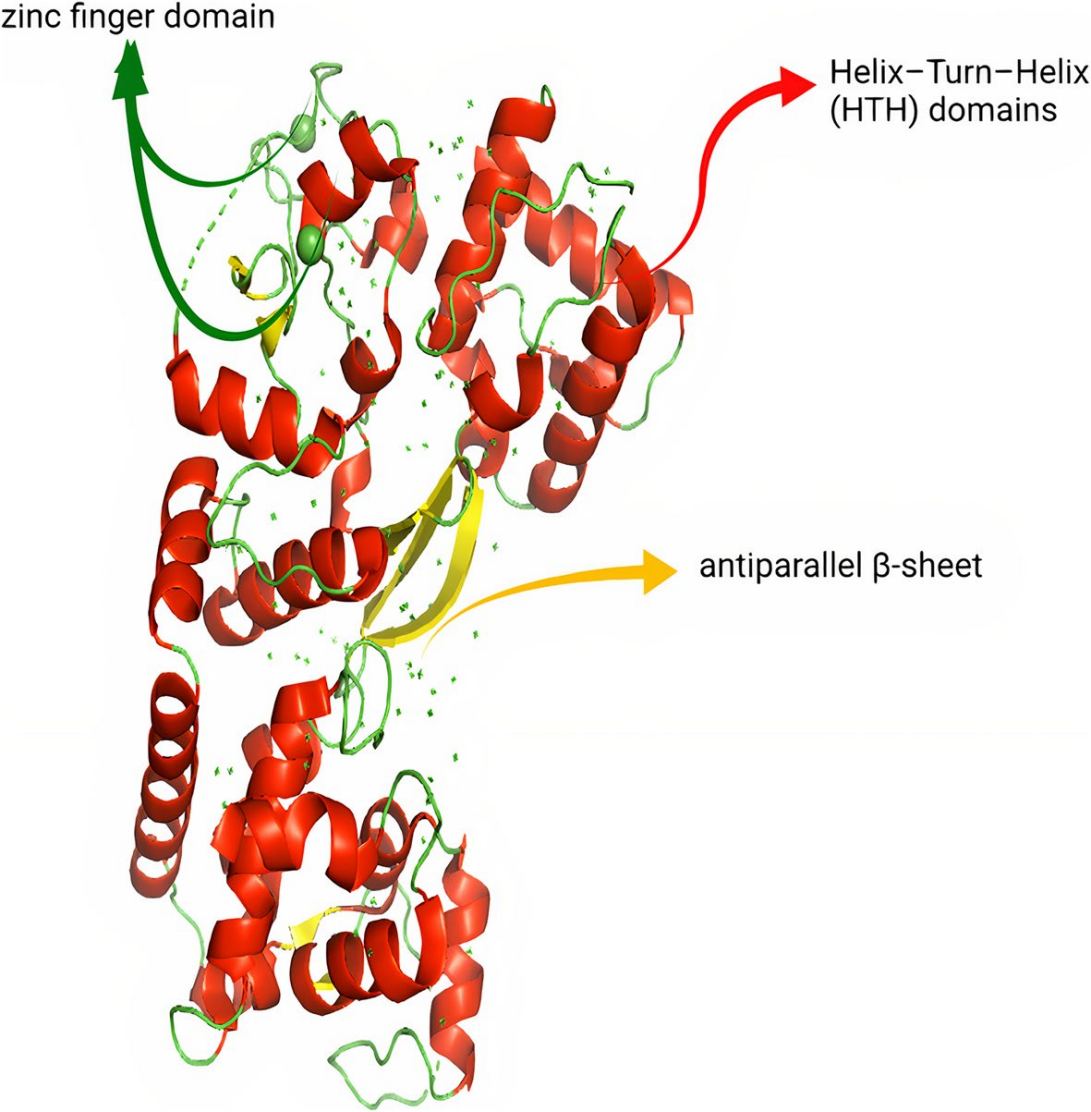
Overall structure of TniQ domains [44]. Adopted from PDB code: 6UVN https://www.rcsb.org/structure/6UVN. The figure was created with PyMol software [55]

TniQ is homologous to the TnsD protein [40, 56, 57]. TniQ is an essential transposition protein located within the Cas operon and is linked to functioning with Cas proteins to guide the transposon to be integrated into the right location within the genome [12, 15]. According to experimental studies, TniQ exists as a homodimer [45]. The N-terminal domain of TniQ is composed of three short α-helices containing helix–turn–helix (HTH) domains, three antiparallel β-sheets, and a zinc finger domain type (CCCH) [15]. The C-terminal domain is composed of ten different length α-helices, a helical domain (HD), a second HTH domain, and two zinc finger motifs [15], as shown in Fig. 6. The presence of the zinc finger domain facilitates protein folding, assembly, recognition of target DNA, and recruitment of TnsC, TnsA, and TnsB [44, 58].

#### TniQ Dimerization and Binding to the Cascade/crRNA Complex

**Fig. 7:**
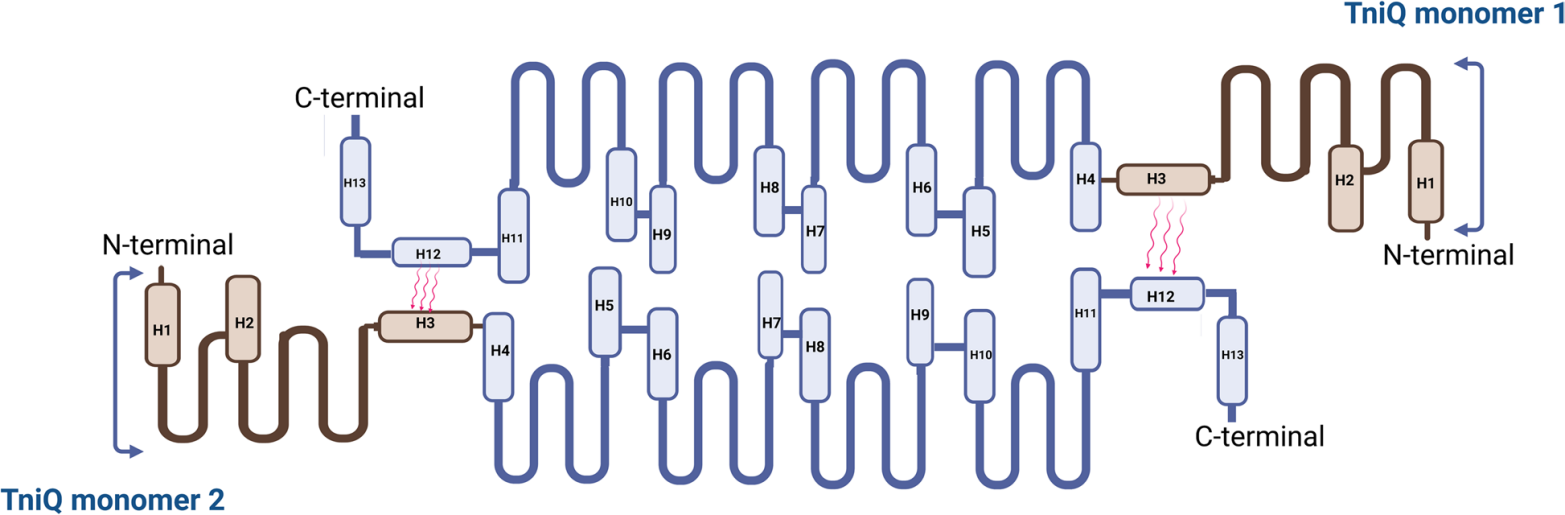
Head-to-tail TniQ dimerization: The C-terminal domain is made up of ten α-helices, whereas the N-terminal domain is made up of three α-helices. One TniQ monomer binds to the other TniQ monomer via hydrophilic interactions between α-helix 3 and α-helix 12 [15]. Created with BioRender.com

Cryo-EM of the binary complex (Cascade/crRNA-TniQ) revealed that the cascade complex contains a head-to-tail TniQ dimer, with one TniQ monomer bound to the other via hydrophilic interactions, specifically between α-helix 3 and α-helix 12, as shown in Fig. 7 [15, 42, 44, 45]. The C-terminal domain of the first TniQ monomer interacts with Cas6, whereas the N-terminal domain of the second TniQ monomer interacts with Cas7.1 [15, 42, 44, 45]. Structural studies of the apo-TniQ and TniQ-bound cascade showed that the Cas7 backbone and Cas8 are almost identical (except for the Cas8 helical bundle (HB), in which TniQ binding to the cascade complex moves the Cas6 away from Cas8HB [44]. A minor conformational change was observed between the TniQ dimer and apo-TniQ, where the loop interacting with Cas6 becomes ordered in addition to a slight movement in the helix interacting with Cas7.1 [42].

4. Binding of the Cascade/crRNA-TniQ Complex to DNA Target.

In the *Vibrio cholera* cascade (VCasacde), Cas8 recognizes PAM sequences in double-stranded target DNA, leading to DNA unwinding and the onset of R-loop formation [15, 42, 44, 45]. The Cas8 protein consists of three domains: the N-terminal domain (Cas8NTD), middle helical bundle (Cas8HB), and C-terminal domain (Cas8CTD) [15, 42, 44, 45]. The Cas8 helical bundle (Cas8HB) probably has no role in the PAM recognition process because its location within Cas8 is distal. Still, it is expected to trigger the activation of TniQ to initiate the DNA transposition process [15, 42, 44, 45]. The integration efficiency was tested for dinucleotide PAM sequences other than the original 5′-CC-3′ PAM system in the INTEGRATE system [12]. All mutants with 5′-CN-3′ PAM showed indistinguishable integration efficiency compared to the original PAM, indicating PAM recognition flexibility in the INTEGRATE system [12]. The arginine residue of Cas8 forms a stacking interaction with the guanine nucleotide on the target strand, which allows the positively charged arginine residue to act as a wedge to separate double-stranded DNA [15, 42, 44, 45]. The separation of double-stranded DNA allows crRNA to invade and base pairs with the target strand while displacing the non-target strand to form the R-loop structure [15, 42, 44, 45]. R-loop formation starts within the first eight PAM-proximal nucleotides of the crRNA, known as seed sequences; then the base pairing will extend, in which the entire spacer region of the crRNA base pairs with the protospacer part of the target DNA [15]. In the INTEGRATE system, the R-loop structure is one of the proofreading checkpoints that direct DNA transposition to a specific site in the genome [15]. Notably, the binding to the DNA indices minimal conformational changes in the cascade/crRNA-TniQ complex, mainly because of a slight opening of the complex and a slight increase in crRNA length [42, 45].

5. TniQ is Expected to Recruit the TnsC, TnsA, and TnsB to Perform the Transposition Process:

TniQ binds to the PAM distal end of the DNA-bound cascade complex and is expected to recruit Tn6677-DNA-loaded TnsABC to initiate transposition [15, 42, 44, 45]. Protein–protein interaction, such as the interaction between TniQ and TnsC, TnsC and TnsA/B, and TnsA and TnsB, is required to perform Tn7 transposition [15, 42, 44, 45]. Further experimental studies are necessary to confirm this interaction network using the INTEGRATE system. The following section describes the structure and function of each transposition protein (TnsC, TnsB, and TnsC) and the experimentally validated interactions between them.

### The AAA + Domain of TnsC Mediates the Interaction with TniQ, TnsA, and TnsB

Structural analysis of TnsC shows that it is a multidomain protein consisting of 555 amino acids, with Walker A and B motifs involved in DNA binding [59]. TnsC is an ATPase-associated activity (AAA) protein that acts as a switch (on or off) for Tn7 transposition [30, 59]. Tn7 transposition does not occur in the presence of ADP; TnsC hydrolyzes ADP to ATP to perform the transposition process [57, 60]. The ATPase activity of TnsC is weak; in the absence of TnsD or TnsE, TnsC cannot participate in transposition unless it is activated by gain-of-function mutations [57, 60]. The N-terminal region of the AAA + ATPase domain is necessary for interactions with TnsD, whereas the C-terminal region is necessary for interactions with both TnsA and TnsB [43, 61]. To activate the transposition process, TnsC must interact directly with the target DNA [43, 56]. When TnsD binds to its attachment site (attTn7), it introduces a conformational change or DNA distortion in the minor groove of the target DNA to introduce a binding site for TnsC [56, 62]. A recent cryo-EM structure showed that TnsC forms open rings on the target DNA, stabilizing due to the interaction between TnsC and TnsD [43]. Another recent Cryo-EM structure showed the interaction between the TniQ monomer and TnsC in the Type V-K CRISPR-Cas System linked to the Tn7-like family (shCAST) [63]. In this structure, TnsC, in the presence of ATP, surrounds one of the DNA strands with continuous filaments, transmitting information to transposases (TnsA and TnsB) to perform the transposition process [63]. This process propagates until TniQ caps the TnsC filaments [63]. Following the interaction between TnsD and TnsC, TnsC introduces another conformational change in the target DNA to make the major groove available for transposases TnsA and TnsB [40, 56]. As mentioned previously, the C-terminal region of TnsC interacts with both TnsA and TnsB, and the C-terminal region of the TnsC ring reorients itself to promote the interaction between TnsC and transposes (TnsA and TnsB) [43]. TnsC can also inhibit transposition to the site already occupied by the Tn7 transposon in a process called target immunity [30, 64, 65]. Once TnsC recruits TnsB, the latter leads to the disassembly of TnsC upon ATP hydrolysis to avoid re-transposition in the already occupied sits [43]. It should be noted that the interaction between TniQ and TnsC could be different in INTEGRATE because TniQ is a dimer in the INTEGRATE system [63].

### Interaction between TnsC and the Transposases (TnsA/TnsB) is Crucial for DNA Binding

TnsA consists of 273 amino acids arranged into two domains, the N-terminal domain characterized by (seven α helices, α1–α7) and (eight β sheets, β1–β8), similar to type II restriction endonuclease enzymes such as the FokI restriction enzyme [66]. α7 is connected to the C-terminal domain, which is characterized by (two alpha helicases, α8–α10, and two beta sheets, β9-β10); it contains a helix–turn–helix (HTH) located within α9 and α10 [66, 67]. TnsA binds to DNA nonspecifically, while TnsB has site-specific DNA binding activity, which explains why TnsA relies on TnsB for DNA recognition and binding [38]. The N-terminal domain of TnsA contains active site residues of TnsA with two bound magnesium ions and one chloride ion [66]. The presence of Mg and Cl ions is expected to provide charge balance and participate in electrostatic interactions [66]. The interaction between TnsC and TnsA is essential for DNA binding, and it has been proposed that TnsA alone does not bind to DNA until it associates with TnsC [61]. Notably, the gain- and loss-of-function mutations allow TnsA transposition without the association of TnsC [60, 61]. The binding of different versions of TnsC to TnsA has been experimentally confirmed [61]. Full-length TnsC (residues 1–555) and the truncated version that has only a C-terminal domain (residues 294–555) [61, 68]. Upon TnsA-TnsC complex formation, most of the interactions (80%) were hydrophobic, while the remaining interactions were polar, with only two salt bridges present at the interface [61]. As mentioned earlier, there are two magnesium ions around the active site of apo-TnsA; however, only one magnesium ion is present when TnsA is bound to TnsC, while the second magnesium ion dissociates owing to TnsC binding [61].

TnsB (702-aa) belongs to the transposase-retroviral integrase superfamily, characterized by the presence of the catalytic triad of Asp, Asp, and Glu (DDE) in the active site contributes to the cleavage and joining activities; in addition to the presence of zinc-binding motif (type HH-CC) contributes to DNA binding [69–73]. The C-terminal domain of TnsB interacts with the C-terminal domain of TnsC [74]. Mutation of TnsC, in particular, TnsC residues (Leu 475 and Leu 476), prevents the ability of TnsC to interact with TnsB and prevent Tn7 transposition, which indicates that these two amino acids are key residues that promote TnsC-TnsB binding [74]. TnsC interaction with transposases (TnsA and TnsB) orient both the donor DNA and target DNA in the vicinity to activate the function of the transposases (TnsA and TnsB) [61].

### TnsA and TnsB Work together to excise the Tn7 Transposon from one site and insert it into the new site within the genome

TnsA and TnsB collaborate to completely excise the Tn7 transposon from its original site; TnsA cleaves the 5′ ends of the Tn7 transposon, while TnsB cleaves the 3′ ends of the Tn7 transposon, then, TnsB joins the excised element to the target DNA by mediating a subsequent DNA strand transfer reaction [73, 75–78]. TnsA may be recruited to the ends of the Tn7 transposon by interacting with TnsB, and then TnsA acts a second time to join the two ends of the Tn7 transposon [79, 80]. Breakage and joining events occur only in the presence of both TnsA and TnsB, in which TnsA lacks DNA-specific binding activity and relies on TnsB to bind DNA [79, 80].

6. The cascade/crRNA-TniQ-TnsABC complex inserts the Tn6677 transposon in the DNA region ~ 50 bp downstream of the PAM site.

The DNA integration process in the INTEGRATE system is very precise, occurring at a fixed location downstream of the PAM binding site [12]. A 95% DNA insertion occurs–48–50 bp from the cascade target site, indicating that the distance from the cascade binding site determines the specific insertion point [12]. Integration may occur in two possible orientations; interestingly, both occurred at a fixed distance downstream of the cascade target site [12].

### Mutations affecting the DNA binding, the TniQ Dimerization, and the integration efficiency

Mutation of the TniQ domain residues involved in ionic interactions (Valine 267 and Valine 268) to glycine reduced the binding affinity between VcCascade and TniQ, leading to a deficiency in DNA integration [44]. In comparison, the mutations E88R, R96E, E379R, and R387E in TniQ inhibit dimer formation and lead to a deficiency in DNA integration [45]. The effect of mutating the length of the spacers in crRNAs by expanding or shortening the spacer length was tested, in which the crRNAs with spacers shortened by 6-nucleotide increments from the 3′ end showed little or no effect on DNA transportation [12]. In contrast, extending the spacer length facilitated targeted integration but at a reduced level compared to the wild 32-nucleotide spacer [12]. Mutational studies on the Tn7 transposon, including the truncation of one end or both ends of the transposon, have shown that truncated transposons with only one right end change RNA-guided DNA integration orientation, making the integration more efficient [12]. Mutational studies on TnsD (TniQ homologues) involve removing the zinc finger domain that still binds DNA, which suggests multiple DNA binding motifs in addition to the zinc finger in both TnsD (TniQ) [40]. Changing the sequence around the TnsD-binding site in the target DNA causes DNA deformation and reduces transposition by reducing the binding affinity of TnsD and TnsC [56], as shown in Table 4.

**Table 4:**
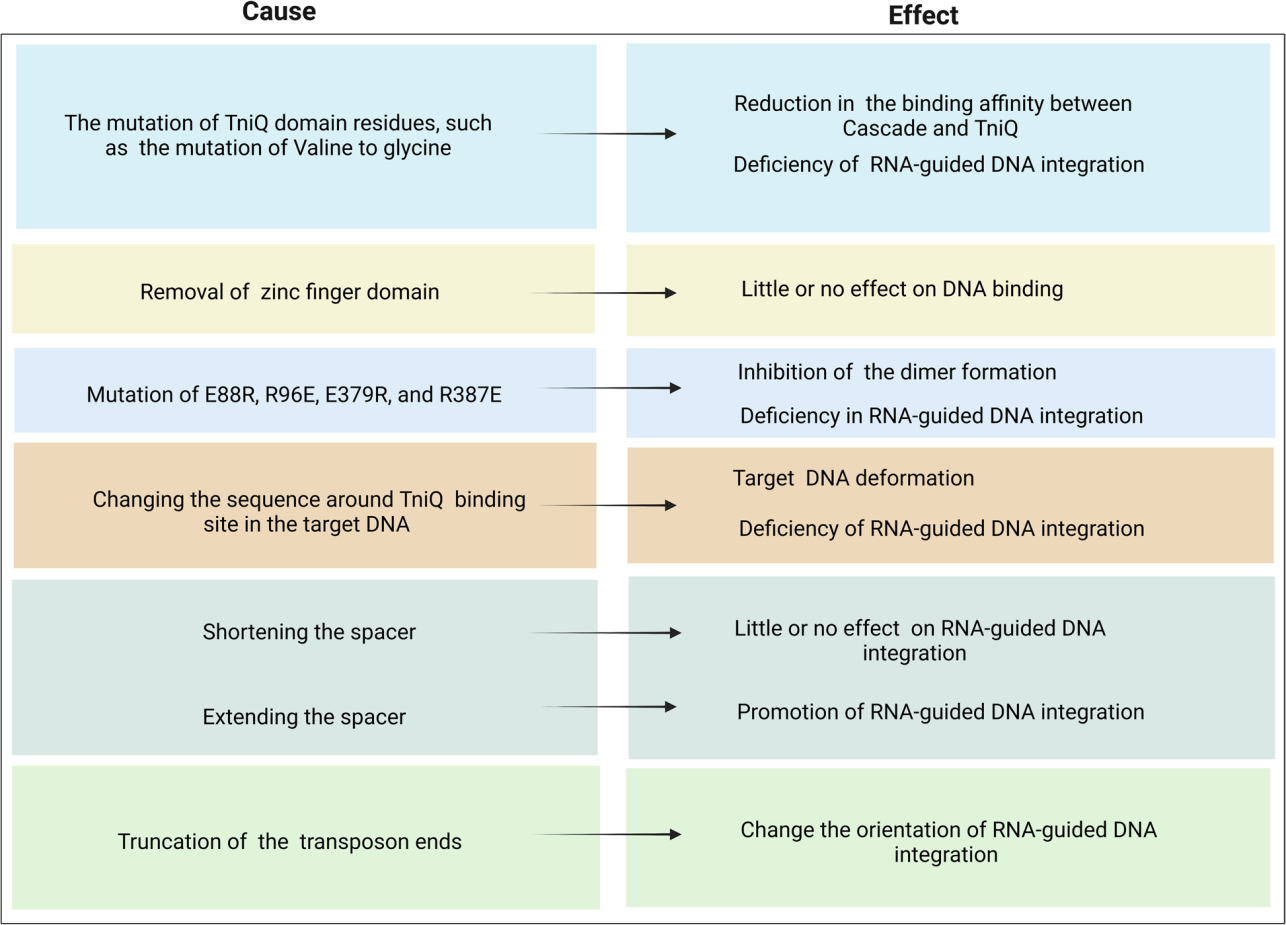
Describes the impact of various mutations on the target DNA binding and the integration efficiency. Created with BioRender.com

The impact of mismatches between the spacer region of the crRNA and the protospacer region of DNA at different regions has been tested [12]. The binding of crRNA to the target DNA is very crucial within the first eight nucleotides "seed sequence "proximal to PAM; the mutation of the seed sequence affects the binding affinity of cascade/crRNA-TniQ to the target, affects the stability of R-loop formation, and most importantly, reduces the DNA transposition [12, 19, 21]. On the other hand, it was proposed that PAM distal mismatch is tolerated; however, recent studies found that PAM distal mismatches at positions 25–28 of the R-loop completely inhibit DNA transposition [12].

### Limitations and Recent Improvements in the Transposons encoded CRISPR-Cas system

**Fig. 8:**
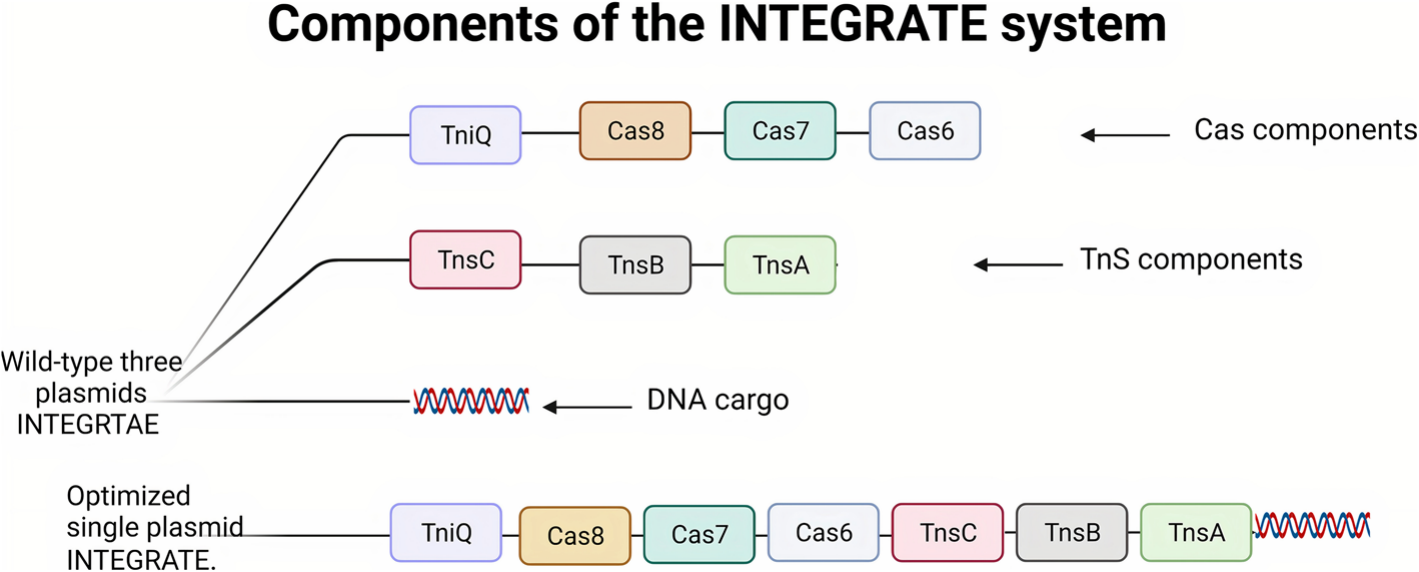
Components of the INTEGRATE System. The wild type INTEGRATE system encodes components in three plasmids. The optimized INTEGRATE encodes all components in a single plasmid [48]. Created with BioRender.com

It is important to note that multiple components in both Tn7 -Encoded Type I-F and V-K CRISPR-Cas systems limit their applications [12, 13, 81]. Discovering or engineering new variants of CRISPR transposon systems with fewer components will significantly enhance the utility of the transposon-encoded CRISPR-Cas system as a gene-editing tool. Researchers recently designed a fusion of *V. cholerae* TnsA and TnsB, which has wild-type integration efficiency, but with fewer components than the wild-type INTEGRATE system [48], as shown in Fig. 8. Another recent successful trial optimized the INTEGRATE system by encoding all components required for transposition in a single vector, including guide RNA, Cas components, Tns components, and DNA cargo [81].

Moreover, a new method called Environmental Transformation Sequencing (ET-Seq) has been developed to identify genetically tractable bacteria within complex microbial communities. This method involves the use of DNAediting-tools, such as CRISPR-Cas Transposase (DART) systems, to achieve targeted genome editing of specific organisms within the community. These findings represent a new way to study microbes with broad applications in human, environmental, and industrial microbiomes [82].

## Conclusions

Tn7-like transposons are naturally linked to minimal CRISPR-Cas systems that use a transposition protein known as TniQ to guide crRNA to the target site in DNA, facilitating Tn7 transposition into a new site. The synergy between Tns proteins and Cas genes allows DNA integration to occur at a specific location in DNA. This new system can be programmed to direct the site-specific insertion of DNA sequences to any location. The main advantage of transposon-encoded-CRISPR/Cas systems is that they do not introduce a double-strand break in the DNA, elimination a need for the repair mechanisms, decreasing the chance of introducing underinsured mutations.

Transposon-encoded CRISPR-Cas systems may open new avenues for applications side-stepping the problems of conventional CRISPR-Cas systems. However, extensive investigation is needed to describe the transposition mechanism and post-transposition effects of the transposon-encoded CRISPR-Cas system. Understanding the mechanism of spacer acquisition in the absence of adaptative genes would be crucial. Another point to consider is the effect of DNA–crRNA mismatches on transposition efficiency.

## Data Availability

Not applicable.

## Abbreviations

CRISPR: Clusters of Regularly Interspaced Short Palindromic Repeats
TN6677: A transposon from *Vibrio cholerae* strain
Tn5053: A transposon from *Scytonema hofmannii* strain
CrRNA: The CRIPSR-RNA
Cas1, Cas2, Cas3, Cas4, Cas5, Cas6, Cas7, Cas8, Cas9, Cas10, Cas12, Cas13: CRISPR/Cas-system associated proteins
TnsA, TnsB, TnsC, TnsD, TnsE, TniQ: Transposon Tn7 transposition protein
PAM: Protospacer Adjacent Motif
DSB: Double-Strand Breaks
attTn7: The Tn7 attachment site
Cascade: CRISPR-associated complex for antiviral defense
VcCascade: *Vibrio-cholera* Cascade complex
INTEGRATE: INsert Transposable Elements by Guide RNA-Assisted Targeting
ShCAST or CAST: CRISPR-associated transposase from cyanobacteria Scytonema hofmanni
Bp: Base-pair
AAA: ATPase Associated Activity
HTH: Helix-Turn-Helix domain

## References

[1] Jansen R, Van Embden JDA, Gaastra W, Schouls LM. Identification of genes that are associated with DNA repeats in prokaryotes. Mol Microbiol 2002;43. 10.1046/j.1365-2958.2002.02839.x. 10.1046/j.1365-2958.2002.02839.x 11952905

[2] Mojica FJM, Díez-Villaseñor C, Soria E, Juez G. Biological significance of a family of regularly spaced repeats in the genomes of Archaea, Bacteria and mitochondria. Mol Microbiol 2000;36. 10.1046/j.1365-2958.2000.01838.x. 10.1046/j.1365-2958.2000.01838.x 10760181

[3] Makarova KS, Wolf YI, Koonin E V. Classification and Nomenclature of CRISPR-Cas Systems: Where from Here? Cris J 2018;1. 10.1089/crispr.2018.0033. 10.1089/crispr.2018.0033 PMC6636873 31021272

[4] Makarova KS, Wolf YI, Alkhnbashi OS, Costa F, Shah SA, Saunders SJ, et al. An updated evolutionary classification of CRISPR-Cas systems. Nat Rev Microbiol 2015;13. 10.1038/nrmicro3569. 10.1038/nrmicro3569 PMC5426118 26411297

[5] Jinek M, Chylinski K, Fonfara I, Hauer M, Doudna JA, Charpentier E. A programmable dual-RNA-guided DNA endonuclease in adaptive bacterial immunity. Science. 2012;337(6096):816–21. 10.1126/science.1225829. 10.1126/science.1225829 PMC6286148 22745249

[6] Cong L, Ran FA, Cox D, Lin S, Barretto R, Habib N, et al. Multiplex genome engineering using CRISPR-Cas systems. Science (80- ) 2013;339. 10.1126/science.1231143. 10.1126/science.1231143 PMC3795411 23287718

[7] Hsu PD, Scott DA, Weinstein JA, Ran FA, Konermann S, Agarwala V, et al. DNA targeting specificity of RNA-guided Cas9 nucleases. Nat Biotechnol 2013;31. 10.1038/nbt.2647. 10.1038/nbt.2647 PMC3969858 23873081

[8] Fu Y, Foden JA, Khayter C, Maeder ML, Reyon D, Joung JK, et al. High-frequency off-target mutagenesis induced by CRISPR-Cas nucleases in human cells. Nat Biotechnol 2013;31. 10.1038/nbt.2623. 10.1038/nbt.2623 PMC3773023 23792628

[9] Jasin M, Haber JE. The democratization of gene editing: Insights from site-specific cleavage and double-strand break repair. DNA Repair (Amst) 2016;44. 10.1016/j.dnarep.2016.05.001. 10.1016/j.dnarep.2016.05.001 PMC5529214 27261202

[10] Klompe SE, Sternberg SH (2018). Harnessing “A billion years of experimentation”: The ongoing exploration and exploitation of CRISPR–Cas Immune Systems. Cris J.

[11] Peters JE, Makarova KS, Shmakov S, Koonin EV (2017). Recruitment of CRISPR-Cas systems by Tn7-like transposons. Proc Natl Acad Sci U S A.

[12] Klompe SE, Vo PLH, Halpin-Healy TS, Sternberg SH (2019). Transposon-encoded CRISPR–Cas systems direct RNA-guided DNA integration. Nature.

[13] Strecker J, Ladha A, Gardner Z, Schmid-Burgk JL, Makarova KS, Koonin EV (2019). RNA-guided DNA insertion with CRISPR-associated transposases. Science (80-)..

[14] Kwon JB, Gersbach CA. Jumping at the chance for precise DNA integration. Nat Biotechnol 2019:4–5. 10.1038/s41587-019-0210-3. 10.1038/s41587-019-0210-3 31371821

[15] Halpin-Healy TS, Klompe SE, Sternberg SH, Fernández IS (2020). Structural basis of DNA targeting by a transposon-encoded CRISPR–Cas system. Nature.

[16] Koonin E V, Makarova KS. CRISPR-Cas: an adaptive immunity system in prokaryotes. F1000 Biol Rep 2009;1. 10.3410/b1-95. 10.3410/B1-95 PMC2884157 20556198

[17] van der Oost J, Jore MM, Westra ER, Lundgren M, Brouns SJJ. CRISPR-based adaptive and heritable immunity in prokaryotes. Trends Biochem Sci 2009;34. 10.1016/j.tibs.2009.05.002. 10.1016/j.tibs.2009.05.002 19646880

[18] Hidalgo-Cantabrana C, Goh YJ, Barrangou R. Characterization and Repurposing of Type I and Type II CRISPR–Cas Systems in Bacteria. J Mol Biol 2019;431. 10.1016/j.jmb.2018.09.013. 10.1016/j.jmb.2018.09.013 30261168

[19] Zheng Y, Li J, Wang B, Han J, Hao Y, Wang S (2020). Endogenous Type I CRISPR-Cas: From Foreign DNA Defense to Prokaryotic Engineering. Front Bioeng Biotechnol.

[20] McDonald ND, Regmi A, Morreale DP, Borowski JD, Fidelma BE (2019). CRISPR-Cas systems are present predominantly on mobile genetic elements in Vibrio species. BMC Genomics.

[21] Hille F, Richter H, Wong SP, Bratovič M, Ressel S, Charpentier E (2018). The Biology of CRISPR-Cas: Backward and Forward. Cell.

[22] Brouns SJJ, Jore MM, Lundgren M, Westra ER, Slijkhuis RJH, Snijders APL, et al. Small CRISPR RNAs guide antiviral defense in prokaryotes. Science (80- ) 2008;321. 10.1126/science.1159689. 10.1126/science.1159689 PMC5898235 18703739

[23] Chylinski K, Le Rhun A, Charpentier E. The tracrRNA and Cas9 families of type II CRISPR-Cas immunity systems. RNA Biol 2013;10. 10.4161/rna.24321. 10.4161/rna.24321 PMC3737331 23563642

[24] Fonfara I, Le Rhun A, Chylinski K, Makarova KS, Lécrivain AL, Bzdrenga J, et al. Phylogeny of Cas9 determines functional exchangeability of dual-RNA and Cas9 among orthologous type II CRISPR-Cas systems. Nucleic Acids Res 2014;42. 10.1093/nar/gkt1074. 10.1093/nar/gkt1074 PMC3936727 24270795

[25] Torres-Ruiz R, Rodriguez-Perales S. CRISPR-Cas9: A revolutionary tool for cancer modelling. Int J Mol Sci 2015;16. 10.3390/ijms160922151. 10.3390/ijms160922151 PMC4613301 26389881

[26] Frangoul H, Altshuler D, Cappellini MD, Chen Y-S, Domm J, Eustace BK, et al. CRISPR-Cas9 Gene Editing for Sickle Cell Disease and β-Thalassemia. N Engl J Med 2021;384. 10.1056/nejmoa2031054. 10.1056/NEJMoa2031054 33283989

[27] Shmakov S, Abudayyeh OO, Makarova KS, Wolf YI, Gootenberg JS, Semenova E, et al. Discovery and Functional Characterization of Diverse Class 2 CRISPR-Cas Systems. Mol Cell 2015;60. 10.1016/j.molcel.2015.10.008. 10.1016/j.molcel.2015.10.008 PMC4660269 26593719

[28] Abudayyeh OO, Gootenberg JS, Konermann S, Joung J, Slaymaker IM, Cox DBT, et al. C2c2 is a single-component programmable RNA-guided RNA-targeting CRISPR effector. Science (80- ) 2016;353. 10.1126/science.aaf5573. 10.1126/science.aaf5573 PMC5127784 27256883

[29] Lee JG, Ha CH, Yoon B, Cheong SA, Kim G, Lee DJ, et al. Knockout rat models mimicking human atherosclerosis created by Cpf1-mediated gene targeting. Sci Rep 2019;9. 10.1038/s41598-019-38732-2. 10.1038/s41598-019-38732-2 PMC6385241 30796231

[30] Peters JE. Tn7 30. Microbiol Spectr 2014;2. 10.1128/microbiolspec. MDNA3–0010–2014.

[31] Dieci G, Fiorino G, Castelnuovo M, Teichmann M, Pagano A. The expanding RNA polymerase III transcriptome. Trends Genet 2007;23. 10.1016/j.tig.2007.09.001. 10.1016/j.tig.2007.09.001 17977614

[32] Gringauz E, Orle KA, Waddell CS, Craig NL. Recognition of Escherichia coli attTn7 by transposon Tn7: lack of specific sequence requirements at the point of Tn7 insertion. J Bacteriol 1988;170. 10.1128/jb.170.6.2832-2840.1988. 10.1128/jb.170.6.2832-2840.1988 PMC211210 2836374

[33] McKown RL, Orle KA, Chen T, Craig NL. Sequence requirements of Escherichia coli attTn7, a specific site of transposon Tn7 insertion. J Bacteriol 1988;170. 10.1128/jb.170.1.352-358.1988. 10.1128/jb.170.1.352-358.1988 PMC210649 2826397

[34] Lichtenstein C, Brenner S. Unique insertion site of Tn7 in the E. coli chromosome. Nature 1982;297. 10.1038/297601a0. 10.1038/297601a0 6283361

[35] Waddell CS, Craig NL. Tn7 transposition: two transposition pathways directed by five Tn7-encoded genes. Genes Dev 1988;2. 10.1101/gad.2.2.137. 10.1101/gad.2.2.137 2834269

[36] Wolkow CA, DeBoy RT, Craig NL. Conjugating plasmids are preferred targets for Tn7. Genes Dev 1996;10. 10.1101/gad.10.17.2145. 10.1101/gad.10.17.2145 8804309

[37] Haren L, Ton-Hoang B, Chandler M. Integrating DNA: Transposases and retroviral integrases. Annu Rev Microbiol 1999;53. 10.1146/annurev.micro.53.1.245. 10.1146/annurev.micro.53.1.245 10547692

[38] Sarnovsky RJ, May EW, Craig NL. The Tn7 transposase is a heteromeric complex in which DNA breakage and joining activities are distributed between different gene products. EMBO J 1996;15. 10.1002/j.1460-2075.1996.tb01024.x. PMC452457 8947057

[39] Parks AR, Peters JE. Tn7 elements: Engendering diversity from chromosomes to episomes. Plasmid 2009;61. 10.1016/j.plasmid.2008.09.008. 10.1016/j.plasmid.2008.09.008 PMC2614081 18951916

[40] Mitra R, McKenzie GJ, Yi L, Lee CA, Craig NL (2010). Characterization of the TnsD-attTn7 complex that promotes site-specific insertion of Tn7. Mob DNA.

[41] Parks AR, Peters JE. Transposon Tn7 is widespread in diverse bacteria and forms genomic islands. J Bacteriol 2007;189. 10.1128/JB.01536-06. 10.1128/JB.01536-06 PMC1855776 17194796

[42] Jia N, Xie W, de la Cruz MJ, Eng ET, Patel DJ (2020). Structure–function insights into the initial step of DNA integration by a CRISPR–Cas–Transposon complex. Cell Res.

[43] Shen Y, Gomez-Blanco J, Petassi MT, Peters JE, Ortega J, Guarné A (2022). Structural basis for DNA targeting by the Tn7 transposon. Nat Struct Mol Biol.

[44] Li Z, Zhang H, Xiao R, Chang L (2020). Cryo-EM structure of a type I-F CRISPR RNA guided surveillance complex bound to transposition protein TniQ. Cell Res.

[45] Wang B, Xu W, Yang H (2020). Structural basis of a Tn7-like transposase recruitment and DNA loading to CRISPR-Cas surveillance complex. Cell Res.

[46] Vo PLH, Ronda C, Klompe SE, Chen EE, Acree C, Wang HH (2021). CRISPR RNA-guided integrases for high-efficiency, multiplexed bacterial genome engineering. Nat Biotechnol.

[47] Liu TY, Doudna JA (2020). Chemistry of Class 1 CRISPR-Cas effectors: Binding, editing, and regulation. J Biol Chem.

[48] Vo PLH, Acree C, Smith ML, Sternberg SH (2021). Unbiased profiling of CRISPR RNA-guided transposition products by long-read sequencing. Mob DNA.

[49] Rybarski JR, Hu K, Hill AM, Wilke CO, Finkelstein IJ (2021). Metagenomic discovery of CRISPR-associated transposons. Proc Natl Acad Sci U S A.

[50] Mohanraju P, Makarova KS, Zetsche B, Zhang F, Koonin E V., Van Der Oost J. Diverse evolutionary roots and mechanistic variations of the CRISPR-Cas systems. Science (80- ) 2016;353. 10.1126/science.aad5147. 10.1126/science.aad5147 PMC13189112 27493190

[51] Charpentier E, Richter H, van der Oost J, White MF. Biogenesis pathways of RNA guides in archaeal and bacterial CRISPR-Cas adaptive immunity. FEMS Microbiol Rev 2015;39. 10.1093/femsre/fuv023. 10.1093/femsre/fuv023 PMC5965381 25994611

[52] Sternberg SH, Haurwitz RE, Doudna JA. Mechanism of substrate selection by a highly specific CRISPR endoribonuclease. RNA 2012;18. 10.1261/rna.030882.111. 10.1261/rna.030882.111 PMC3312554 22345129

[53] Haurwitz RE, Jinek M, Wiedenheft B, Zhou K, Doudna JA. Sequence- and structure-specific RNA processing by a CRISPR endonuclease. Science (80- ) 2010;329. 10.1126/science.1192272. 10.1126/science.1192272 PMC3133607 20829488

[54] Jore MM, Lundgren M, Van Duijn E, Bultema JB, Westra ER, Waghmare SP, et al. Structural basis for CRISPR RNA-guided DNA recognition by Cascade. Nat Struct Mol Biol 2011;18. 10.1038/nsmb.2019. 10.1038/nsmb.2019 21460843

[55] DeLano WL. The PyMOL Molecular Graphics System, Version 2.3. Schrödinger LLC 2020.

[56] Wiedenheft B, Lander GC, Zhou K, Jore MM, Brouns SJJ, Van Der Oost J, et al. Structures of the RNA-guided surveillance complex from a bacterial immune system. Nature 2011;477. 10.1038/nature10402. 10.1038/nature10402 PMC4165517 21938068

[57] Kuduvalli PN, Rao JE, Craig NL (2001). Target DNA structure plays a critical role in Tn7 transposition. EMBO J.

[58] Bainton RJ, Kubo KM, Feng J nong, Craig NL. Tn7 transposition: Target DNA recognition is mediated by multiple Tn7-encoded proteins in a purified in vitro system. Cell 1993;72. 10.1016/0092-8674(93)90581-A. 10.1016/0092-8674(93)90581-a 8384534

[59] Laity JH, Lee BM, Wright PE. Zinc finger proteins: New insights into structural and functional diversity. Curr Opin Struct Biol 2001;11. 10.1016/S0959-440X(00)00167-6. 10.1016/s0959-440x(00)00167-6 11179890

[60] Flores C, Qadri MI, Lichtenstein C. DNA sequence analysis of five genes; tnsA, B, C, D and E, required for Tn7 transposition. Nucleic Acids Res 1990;18. 10.1093/nar/18.4.901. 10.1093/nar/18.4.901 PMC330344 2156235

[61] Stellwagen AE, Craig NL (1997). Gain-of-function mutations in TnsC, an ATP-dependent transposition protein that activates the bacterial transposon Tn7. Genetics.

[62] Ronning DR, Li Y, Perez ZN, Ross PD, Hickman AB, Craig NL (2004). The carboxy-terminal portion of TnsC activates the Tn7 transposase through a specific interaction with TnsA. EMBO J.

[63] Rao JE, Miller PS, Craig NL. Recognition of triple-helical DNA structures by transposon Tn7. Proc Natl Acad Sci U S A 2000;97. 10.1073/pnas.080061497. 10.1073/pnas.080061497 PMC18120 10737770

[64] Park JU, Tsai AWL, Mehrotra E, Petassi MT, Hsieh SC, Ke A (2021). Structural basis for target site selection in RNA-guided DNA transposition systems. Science (80-)..

[65] Robinson MK, Bennett PM, Richmond MH. Inhibition of TnA translocation by TnA. J Bacteriol 1977;129. 10.1128/jb.129.1.407-414.1977. 10.1128/jb.129.1.407-414.1977 PMC234940 318647

[66] Arciszewska LK, Drake D, Craig NL. Transposon Tn7. cis-Acting sequences in transposition and transposition immunity. J Mol Biol 1989;207. 10.1016/0022-2836(89)90439-7. 10.1016/0022-2836(89)90439-7 2544738

[67] Hickman AB, Li Y, Mathew SV, May EW, Craig NL, Dyda F (2000). Unexpected structural diversity in DNA recombination: The restriction endonuclease connection. Mol Cell.

[68] Harrison SC, Aggarwal AK. DNA recognition by proteins with the helix-turn-helix motif. Annu Rev Biochem 1990;59. 10.1146/annurev.bi.59.070190.004441. 10.1146/annurev.bi.59.070190.004441 2197994

[69] Stellwagen AE, Craig NL. Analysis of gain-of-function mutants of an ATP-dependent regulator of Tn7 transposition. J Mol Biol 2001;305. 10.1006/jmbi.2000.4317. 10.1006/jmbi.2000.4317 11152618

[70] Hare S, Gupta SS, Valkov E, Engelman A, Cherepanov P. Retroviral intasome assembly and inhibition of DNA strand transfer. Nature 2010;464. 10.1038/nature08784. 10.1038/nature08784 PMC2837123 20118915

[71] Zheng R, Jenkins TM, Craigie R. Zinc folds the N-terminal domain of HIV-1 integrase, promotes multimerization, and enhances catalytic activity. Proc Natl Acad Sci U S A 1996;93. 10.1073/pnas.93.24.13659. 10.1073/pnas.93.24.13659 PMC19383 8942990

[72] Hare S, Maertens GN, Cherepanov P. 3′-Processing and strand transfer catalysed by retroviral integrase in crystallo. EMBO J 2012;31. 10.1038/emboj.2012.118. 10.1038/emboj.2012.118 PMC3395085 22580823

[73] Kvaratskhelia M, Sharma A, Larue RC, Serrao E, Engelman A (2014). Molecular mechanisms of retroviral integration site selection. Nucleic Acids Res.

[74] Dyda F, Hickman AB, Jenkins TM, Engelman A, Craigie R, Davies DR (1994). Crystal structure of the catalytic domain of HIV-1 integrase: Similarity to other polynucleotidyl transferases. Science (80-).

[75] Choi KY, Spencer JM, Craig NL (2014). The Tn7 transposition regulator TnsC interacts with the transposase subunit TnsB and target selector TnsD. Proc Natl Acad Sci U S A.

[76] McKown RL, Waddell CS, Arciszewska LK, Craig NL. Identification of a transposon Tn7-dependent DNA-binding activity that recognizes the ends of Tn7. Proc Natl Acad Sci U S A 1987;84. 10.1073/pnas.84.22.7807. 10.1073/pnas.84.22.7807 PMC299398 2825163

[77] May EW, Craig NL. Switching from cut-and-paste to replicative Tn7 transposition. Science (80- ) 1996;272. 10.1126/science.272.5260.401. 10.1126/science.272.5260.401 8602527

[78] Arciszewska LK, Craig NL (1991). Interaction of the Tn7-encoded transposition protein TnsB with the ends of the transposon. Nucleic Acids Res.

[79] Peters JE, Fricker AD, Kapili BJ, Petassi MT (2014). Heteromeric transposase elements: Generators of genomic Islands across diverse bacteria. Mol Microbiol.

[80] Biery MC, Lopata M, Craig NL. A minimal system for Tn7 transposition: The transposon-encoded proteins TnsA and TnsB can execute DNA breakage and joining reactions that generate circularized Tn7 species. J Mol Biol 2000;297. 10.1006/jmbi.2000.3558. 10.1006/jmbi.2000.3558 10704304

[81] Choi KY, Li Y, Sarnovsky R, Craig NL. Direct interaction between the TnsA and TnsB subunits controls the heteromeric Tn7 transposase. Proc Natl Acad Sci U S A 2013;110. 10.1073/pnas.1305716110. 10.1073/pnas.1305716110 PMC3670325 23674682

[82] Rubin BE, Diamond S, Cress BF, Crits-Christoph A, He C, Xu M, et al. Targeted genome editing of bacteria within microbial communities. BioRxiv 2020:1–49. 10.1101/2020.07.17.209189.

